# Novel oncogenic and chemoresistance-inducing functions of resistin in ovarian cancer cells require miRNAs-mediated induction of epithelial-to-mesenchymal transition

**DOI:** 10.1038/s41598-018-30978-6

**Published:** 2018-08-21

**Authors:** Ling Qiu, Guo-Feng Zhang, Lei Yu, Hong-Yong Wang, Xiao-Jing Jia, Tie-Jun Wang

**Affiliations:** 1 Department of Radiation Oncology, The Second Hospital of Jilin University, Changchun, China; 2 Department of Hepatobiliary and Pancreatic Surgery, The Second Hospital of Jilin University, Changchun, China

## Abstract

Resistin plays a role in the growth, proliferation, angiogenesis, metastasis and therapeutic resistance in different cancers. However, such effects of resistin have never been evaluated in ovarian cancer, a deadly gynecological malignancy. We observed a significant induction of ovarian cancer cells’ growth, invasion and cisplatin resistance, and established a mechanism of resistin action that included induction of EMT and stemness, as evidenced by down-regulated epithelial marker e-cadherin and up-regulated mesenchymal markers vimentin/ ZEB1 and stemness markers sox2, oct4 and nanog. The mechanism also included suppression of tumor suppressor miRNAs, let-7a, miR-200c and miR-186. Over-expression of these miRNAs significantly reversed the resistin-mediated effects on invasion and chemoresistance. We further validated our results *in vivo* where resistin administration significantly enhanced tumor growth in mice. Our results provide first evidence for such oncogenic effects of resistin in ovarian cancer models and a rationale for future studies to further understand the mechanistic role of resistin in ovarian cancer invasiveness, metastasis and therapy resistance.

## Introduction

Ovarian cancer is the deadliest among all gynecological cancers. A major reason for the increased ovarian cancer mortality is its late, advanced stage detection. There is an urgent need to find novel molecular factors responsible for the aggresiveness of ovarian cancers, which can be targeted for therapy. To address this, we focused on resistin, a macrophage-derived cytokine which is related to obesity and has also been studied in relation to insulin resistance. Resistin levels have been associated with high grade cancers and/or relapse free survival in many human cancers, such as breast^1^, chondrosarcoma^2^, colorectal^3,4^, endometrial^5,6^, gastroesophageal^7,8^, lung^9^, multiple myeloma^10^, prostate^11^ and renal^12^ cancers.

Resistin is not only associated with high grade cancers and survival of patients, research in recent years has raised the possibility of resistin playing a role in chemotherapy^13,14^. High levels of resistin correlate with decreased sensitivity to chemotherapy in multiple cancers^15,16^. Further, acquired resistance against chemotherapy is a major clinical challenge for ovarian cancer patients^17^, which underlines the significance of such investigations. Based on the reported involvement of resistin in different cancers, particularly its role in acquired resistance, we hypothesized that resistin might be responsible for similar pro-oncogenic and chemoresistance-inducing functions in ovarian cancer cells as well. We investigated a role of resistin in the growth, clonogenicity, invasion and cisplatin-resistance of ovarian cancer cells, and sought to elucidate the mechanistic details of such resistin-mediated effects. We found that resistin induces epithelial-to-mesenchymal transition (EMT), along with increased stemness and decreased expression of several EMT-regulating miRNAs, which provides the mechanism of its action.

## Materials and Methods

### Cell lines and Reagents

Ovarian cancer cells SK-OV-3 were purchased from ATCC (Manassas, USA) and the A2780 cells, along with their cisplatin-resistant derivative cells (A2780Cis) were purchased from Sigma (St Louis, USA). All cells were cultured in RPMI 1640 media, with the presence of 10% fetal bovine serum, in 5% CO_2_–humidified atmosphere at 37 °C.

### MTT Cell Proliferation Assay

MTT Cell Proliferation Assay kit was obtained from ATCC (Manassas, USA) and the instructions were followed, as supplied by the vendor. The reduction of tetrazolium salts is an established method to quantitate cell proliferation. In this assay, tetrazolium MTT (3-(4, 5-dimethylthiazolyl-2)-2, 5-diphenyltetrazolium bromide) is reduced by cells that are metabolically active, partly due to the action of dehydrogenase enzymes, resulting in generation of reducing equivalents such as NADH and NADPH. Cells were seeded in 96 well plates a night before experiment. Treatments were performed, as indicated in individual figures. At the end of treatment, 10 μl MTT reagent was added on top of the incubated reaction mixtures for 2.5 hours. This was followed by addition of supplied detergent reagent (100 μl) for 4 hours and the colorimetric quantitation of plates at 575 nm in a plate reader.

### Colony-formation assay

We performed anchorage-dependent colony formation assay to assess the effect of resistin on the clonogenic potential of ovarian cancer cells. A2780 and SK-OV-3 cells were collected by trypsinization and resuspended in complete culture medium. Single cell suspensions were seeded in 6-well plates at a density of 750 cells per well overnight and then resistin or the vehicle control were added, as appropriate. After two weeks of growth in an incubator under 5% O_2_, 5% CO_2_ and 90% N_2_ conditions, colonies were fixed, using 4% paraformaldehyde, and then stained with crystal violet. Pictures were taken and the colonies were calculated, using NIH Scion image analysis software.

### Cell invasion assay

Cell invasion assay was performed using 24-well plates with inserts (8 µM pores), in which the inserts were coated with growth factor reduced Matrigel. First, cells were trypsinized and then suspended in serum free RPMI 1640 medium, before seeding on the plates. Bottom of the wells was filled with RPMI 1640 with 10% FBS. At the end of assay, cells that had invaded through matrigel were stained using 4 µg/ml Calcein AM (ThermoFisher Scientific, USA) in PBS for an hour at room temperature and pictures were taken using a fluorescent microscope. Thereafter, cells were recovered from the bottom of inserts by trypsinization and counted, using hemocytometer. Additionally, fluorescence of invading cells was quantitated by collecting all invaded cells from individual test conditions into individual wells of a 96-well plate and evaluating fluorescence using a fluorescence plate reader.

### VEGF and MMP2 ELISA detections

VEGF and MMP2 quantitations was done using specific ELISA kits (R&D Systems, USA). Culture media from cells under different experimental conditions was collected and concentrated. Secreted VEGF/MMP2 was determined by following vendor instructions. The ELISAs were based on quantitative sandwich enzyme immunoassay technique and the assay plates supplied by vendor were coated with appropriate specific antibodies on to which the standards, controls and test samples were added. Incubation was done for 2 hours at room temperature. After three washings with supplied wash buffer, an enzyme-linked polyclonal antibody specific for VEGF or MMP2 (as appropriate) was added for 2 hours at room temperature, followed by further three washings with wash buffer and addition of a substrate (20 minutes at room temperature) that was acted upon by conjugated enzyme resulting in development of color that was quantitated calorimetrically at 450 nm.

### Real-Time qRT-PCR for mRNA detection

RNA was isolated using the Trizol reagent (ThermoFisher Scientific, USA) by following the exact protocol supplied by the vendor. Treatment with 25 ng/mL resistin was for 72 hours. First, trizol reagent (0.8 mL) was added to collected cell pellet, followed by addition of 0.2 mL chloroform and incubation for 5 minutes at room temperature. Samples were then centrifuged at 4 °C at 12,000 × g for 15 minutes. The upper clear aqueous phase was collected and 0.5 mL of isopropanol was added (for 10 minutes) to precipitate RNA. This was followed by centrifugation at 4 °C at 12,000 × g for 10 minutes. Collected RNA pellet was washed with 75% ethanol before centrifugation at 4 °C at 7,500 × g for 5 minutes. Real-time qRT-PCR was performed to quantitate messenger RNA (mRNA) expression, using Applied Biosystems real-time PCR instrument. GAPDH expression was quantitated as internal control. The sequences of all primers used in this study for quantitative RT-PCR of mRNAs are provided in Table 1.

**Table 1:** List of primers used for qRT-PCR in the current study.

	Forward (5′-3′)	Reverse (5′-3′)
E-Cadherin	TGGAGGAATTCTTGCTTTGC	CGTACATGTCAGCCAGCTTC
GAPDH	GGCTGAGAACGGGAAGCTTGTCAT	CAGCCTTCTCCATGGTGGTGAAGA
MMP2	CCCTGATGTCCAGCGAGTG	ACGACGGCATCCAGGTTATC
Nanog	TGGGATTTACAGGCGTGAGCCAC	AAGCAAAGCCTCCCAATCCCAAAC
Oct4	GGGCTCTCCCATGCATTCAAAC	CACCTTCCCTCCAACCAGTTGC
Sox2	CCATGCAGGTTGACACCGTTG	TCGGCAGACTGATTCAAATAATACAG
VEGF	CTACCTCCACCATGCCAAGT	GCAGTAGCTGCGCTGATAGA
Vimentin	TCTCTGAGGCTGCCAACCG	CGAAGGTGACGAGCCATTTCC
ZEB1	GCACCTGAAGAGGACCAGAG	TGCATCTGGTGTTCCATTTT

### Real-Time qRT-PCR for miRNA detection

For miRNA analysis, total RNA was isolated using the mirVana miRNA isolation kit (Ambion, USA), following the protocol of vendor. The levels of miRNAs were determined using miRNA-specific Taqman MGB probes from the Taqman MicroRNA Assay (Applied Biosystems, USA), using Applied Biosystems real-time PCR instrument. The relative amounts of miRNA were normalized to U6.

### Sphere formation assay

Sphere formation assay was conducted to evaluate the effect of resistin on stemness of ovarian cancer cells. A2780 and SK-OV-3 cells were collected by trypsinization and 2,000 cells were added to individual wells of ultra-low attachment 24-well plates (Corning, USA). Spheres were allowed to grow for 7 days in sphere formation medium (1∶1 DMEM/F-12 medium supplemented with B-27 and N-2) and then counted under a microscope.

### Experiments involving transfections

For transfections of pre-miRNAs (let-7a, miR-200c, miR-186 or a combination of all three miRNAs), cells were seeded in six wells plates (4 × 10^5^ cells) overnight. They were then transfected with pre-miRs or miRNA-negative controls (Ambion, USA) at a final concentration of 20 nM, using DharmaFECT1 transfection reagent (Dharmacon, USA). After 72 hours, cells were collected again by trypsinization, counted, re-seeded in 6-well plates and pre-miRs or the negative controls were added for two more rounds of transfections of 72 hours each. The control as well as pre-miRs-transfected cells were first exposed to resistin for 6 hours, followed by cisplatin treatment for 72 hours.

### Western blot analysis

For western blot analysis, cellular protein extracts were prepared in RIPA buffer using normal protocol. Treatment with 25 ng/mL resistin was for 72 hours. Samples were run through polyacrylamide gels under denaturing conditions and transferred to PVDF membranes. Membranes were incubated with primary antibodies (as indicated in figures) overnight in a cold room. All primary antibodies were purchased from Santa Cruz Biotechnology. After overnight incubation in cold room, membranes were washed three times and incubated with secondary antibodies (Sigma) for 1 hour at room temperature, followed by three more washings. Chemiluminescence (ThermoFisher Scientific) was used to detect the protein expression.

### *In vivo* experiments

The protocol for our mice *in vivo* experiments was approved by the Animal Research Ethics Committee at the Jilin University. Further, we confirm that all methods were performed in accordance with the relevant guidelines and regulations. A2780 cells (1.5 × 10^6^) were injected subcutaneously into both flanks of female ICR-NOD/SCID mice (Vital River Laboratories, Co., Ltd., China). The mice were housed in sterilized room and food and water was provided *ad libitum*. Tumors were initially allowed to grow for 3 weeks. Thereafter, resistin, or vehicle control, was administered by intravenous injections at a dose of 400 ng/mouse, three days/week, for 3 weeks. There were 10 mice in each group and all the mice (control and treatment group) were sacrificed at the same time (2 weeks after the end of treatment regime). Tumors were measured using calipers and the volume of tumors in mm^3^ was determined by the formula (width^2^ × length)/2. Tumor tissues were further processed to extract mRNA for evaluation of EMT and stemness markers.

### Statistical analysis

The reported results are representative of at least three independent experiments, with each experiment conducted in triplicate. We used student’s t test to evaluate the level of significant differences between group means, and performed statistical analysis using Prism 5 (GraphPad software). A p value of at least <0.05 was considered significant.

## Results

### Effect of resistin on growth, colony formation and invasion of ovarian cancer cells

**Figure 1:**
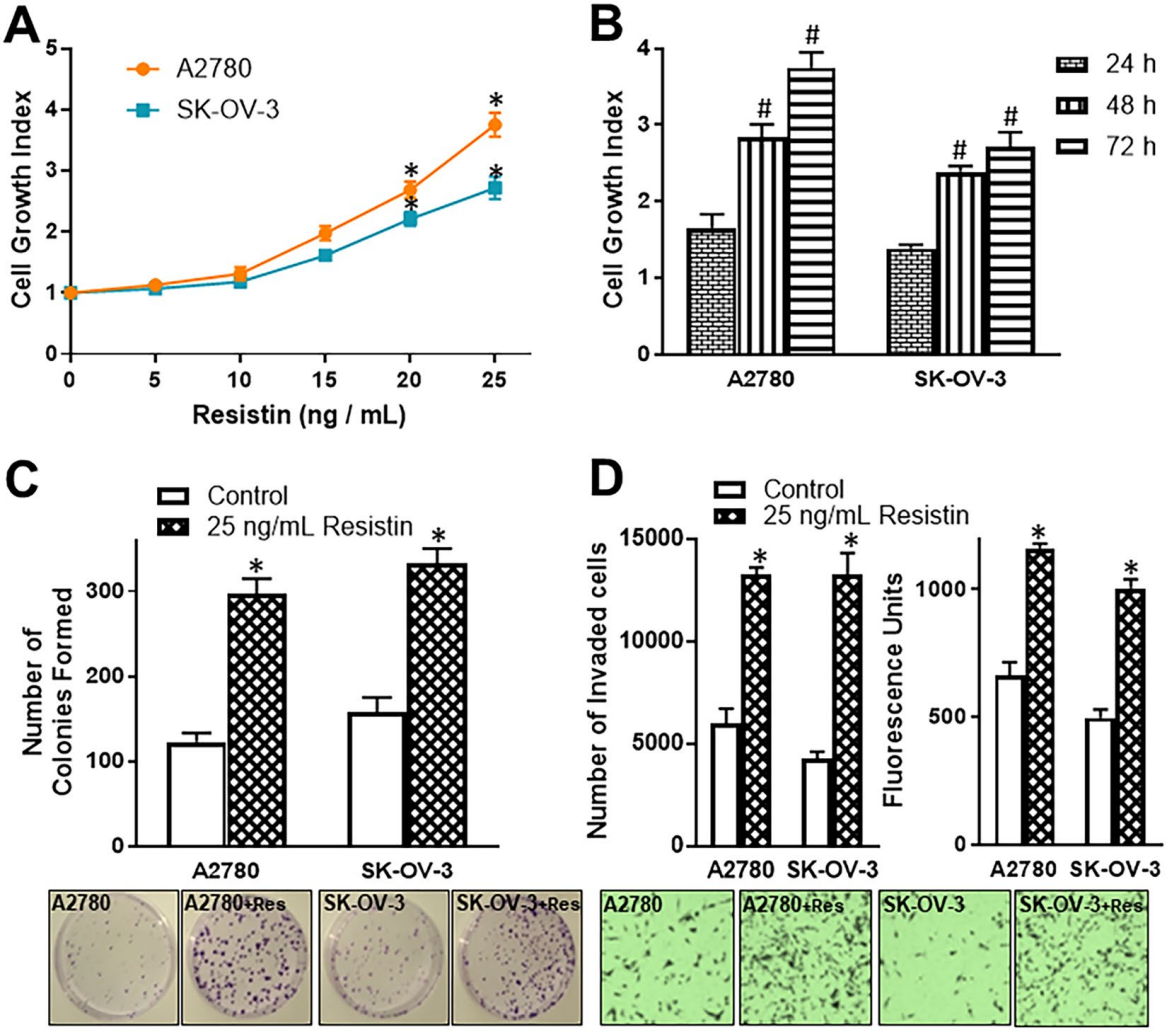
(**A**) Cell growth of ovarian cancer cells A2780 and SK-OV-3 was measured, in the presence of increasing concentrations of resistin, by MTT assay after 72 hours of resistin exposure. The OD value for control cells (0 ng/mL Resistin) was assigned a value of ‘1’ and the relative OD values of resistin-treated cells are plotted to represent cell growth index. (**B**) A time-dependent induction of cell growth was measured by incubating A2780 and SK-OV-3 cells with 25 ng/mL resistin for 24, 48 and 72 hours. The OD value for control cells (0 hours) was assigned a value of ‘1’ and the relative OD values at 24, 48 and 72 hour time-points are plotted to represent cell growth index. Anchorage-dependent colony formation assay (**C**) and invasion assays (**D**) were performed to assess the effects of 25 ng/mL resistin on colony forming ability and invasive potential of A2780 and SK-OV-3, when incubated for 72 hours. For invasion assay, the number of cells that invaded through matrigel were calculated after staining with Calcein AM (left graph) and the fluorescence of collected cells quantitated (right graph). Representative pictures for colonies and invasive cells are shown below the respective bar graphs. ‘+*Res’* in representative pictures indicates assay in the presence of added resistin. *p < 0.01, compared to control and ^#^p < 0.01, compared to cell growth at 24 h.

We started our investigation using two ovarian cancer cell lines, A2780 and SK-OV-3. First, we evaluated the effect of resistin treatment on growth and proliferation of these cells. We treated both of these cell lines with increasing doses of resistin (0, 5, 10, 15, 20 and 25 ng/mL) for 72 hours and observed that resistin significantly stimulates the cell growth (p < 0.01 at 20 and 25 ng/mL doses of resistin) (Fig. 1A). We took the highest dose (25 ng/mL) and performed a time kinetics study. An increase in cell growth, as a function of time of resistin exposure was observed (Fig. 1B). These observations indicated a dose- and time-dependent action of resistin on ovarian cancer cells. Next, we looked at the stimulation of colony formation by resistin, as assessed by anchorage-dependent colony forming assay, and found that 25 ng/mL resistin significantly (p < 0.01) increased the number of colonies in A2780 and SK-OV-3 cells (Fig. 1C). Similarly, resistin also had a significant effect on the invasive potential of A2780 and SK-OV-3 cells (Fig. 1D). The number of cells, that were able to pass through the matrigel-coated wells, was significantly higher (p < 0.01) in resistin-treated A2780 and SK-OV-3 cells, as compared to vehicle-treated control cells (Fig. 1D). This was further confirmed by the fluorescence analyses of detached cells that were stained with Calcein AM (Fig. 1D).

### Resistin induces cisplatin resistance

**Figure 2:**
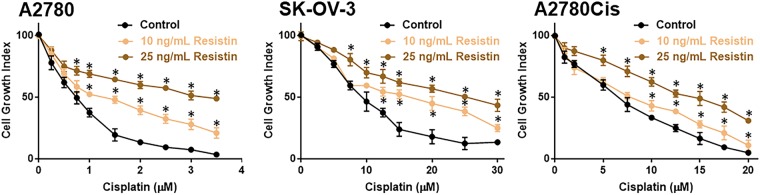
Resistin induces resistance to cisplatin. A2780, SK-OV-3 and A2780Cis cells were treated with increasing doses of cisplatin in the absence and presence of resistin (two doses – 10 and 25 ng/mL) for 72 hours, before being subjected to MTT assay. The OD value for control cells (0 μM cisplatin) was assigned a value of ‘100’ and the relative OD values of cells treated with increasing concentrations of cisplatin are plotted to represent cell growth index. *p < 0.05, compared to control.

It has been suggested that resistin can induce resistance against chemotherapy^13,14^, and therefore, we next evaluated if resistin can, similarly, induce resistance against cisplatin in ovarian cancer cells. We chose to study sensitivity to cisplatin because this is a common chemotherapy used to treat ovarian cancer patients. We performed our experiments by picking two different doses of resistin (10 and 25 ng/mL). A2780 cells were treated with increasing doses of cisplatin for 72 hours either alone (vehicle control) or in the presence of two resistin doses. The lower dose, 10 ng/mL, significantly increased resistance against cisplatin and the higher dose, 25 ng/mL, increased the resistance even further (Fig. 2A). Thus, resistin, dose-dependently, induced resistance against cisplatin. Similar results were observed for SK-OV-3 (Fig. 2B) as well as A2780Cis cells, the cisplatin-resistant derivatives of parental A2780 cells (Fig. 2C). Based on these results, we calculated cisplatin IC-50 values of all the three cell lines tested (Table 2), which revealed ~1.7-folds and ~4.5-folds increase in IC50 of A2780 cells treated with 10 ng/mL and 25 ng/mL resistin, respectively, ~1.2-folds and ~2.2-folds in IC50 of A2780Cis cells and ~1.8 and 2.8-folds increase in IC50 of SK-OV-3 cells (Table 2).

**Table 2:** IC50 values of different ovarian cancer cell lines against cisplatin.

IC50 values (μM)					
A2780		A2780Cis		SK-OV-3	
*Control*	0.74 ± 0.03	*Control*	6.58 ± 0.12	*Control*	8.85 ± 0.21
*10 ng/mL vResistin*	1.28 ± 0.05	*10 ng/mL Resistin*	7.81 ± 0.15	*10 ng/mL Resistin*	16.21 ± 0.35
*25 ng/mL Resistin*	3.36 ± 0.08	*25 ng/mL Resistin*	14.93 ± 0.22	*25 ng/mL Resistin*	25.6 ± 0.56

The data from Fig. 2A–C was used to calculate IC-50 values, and all data represents Mean ± S.E.

### Resistin induces markers of angiogenesis

**Figure 3:**
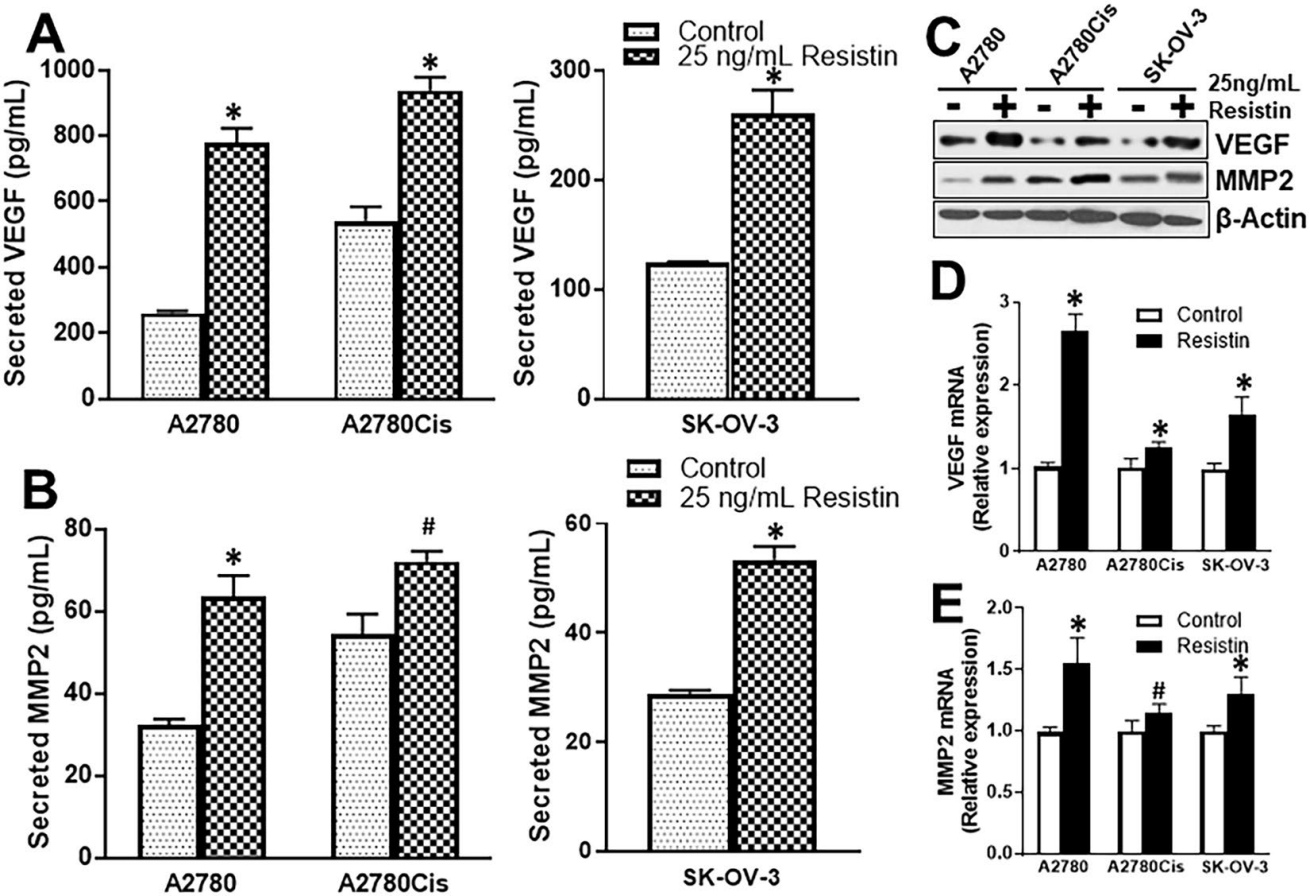
Resistin induces secretion of VEGF and MMP2. (**A**) VEGF and (**B**) MMP2 secretion by parental A2780, cisplatin-resistant A2780Cis and SK-OV-3 cells was quantitated using ELISA. Cell were treated with resistin, (25 ng/mL) or vehicle control for 72 hours. The numbers were normalized to number of living cells, which were collected by trypsinization and subjected to analyses by (**C**) western blotting for evaluation of protein expression and quantitative RT-PCR for evaluation of (**D**) VEGF and (**E**) MMP2 mRNA levels. mRNA levels in control (vehicle-treated) cells were set to a value of 1 and relative levels in resistin-treated cells are shown. β-actin was used as loading control for western blots while GAPDH was used as reference control for RT-PCR analyses. ^#^p < 0.05 and *p < 0.01, compared to control.

Angiogenesis is important for growth and metastasis of cancers. We evaluated secretion of markers of angiogenesis, VEGF and MMP2, by ovarian cancer cells. First, we compared parental A2780 cells and their cisplatin-resistant counterparts. We found that cisplatin resistant A2780Cis cells secreted more VEGF that the parental A2780 cells (Fig. 3A). Further, 25 ng/mL resistin increased this secretion of VEGF in all the ovarian cancer cells tested (Fig. 3A). Similar results were obtained for the other angiogenesis marker (MMP2) as well. Cisplatin-resistant A2780Cis cells secreted higher amounts of MMP2, as detected by ELISA, compared to parental A2780 cells (Fig. 3B), which was induced by resistin in both cells as well as in SK-OV-3 cells (Fig. 3B). Furthermore, western blot (Fig. 3C) and quantitative RT-PCR analysis further confirmed the findings that 25 ng/mL resistin induced VEGF (Fig. 3D) and MMP2 (Fig. 3E), at both mRNA transcript and protein expression levels.

### Resistin induces EMT and stemness

**Figure 4:**
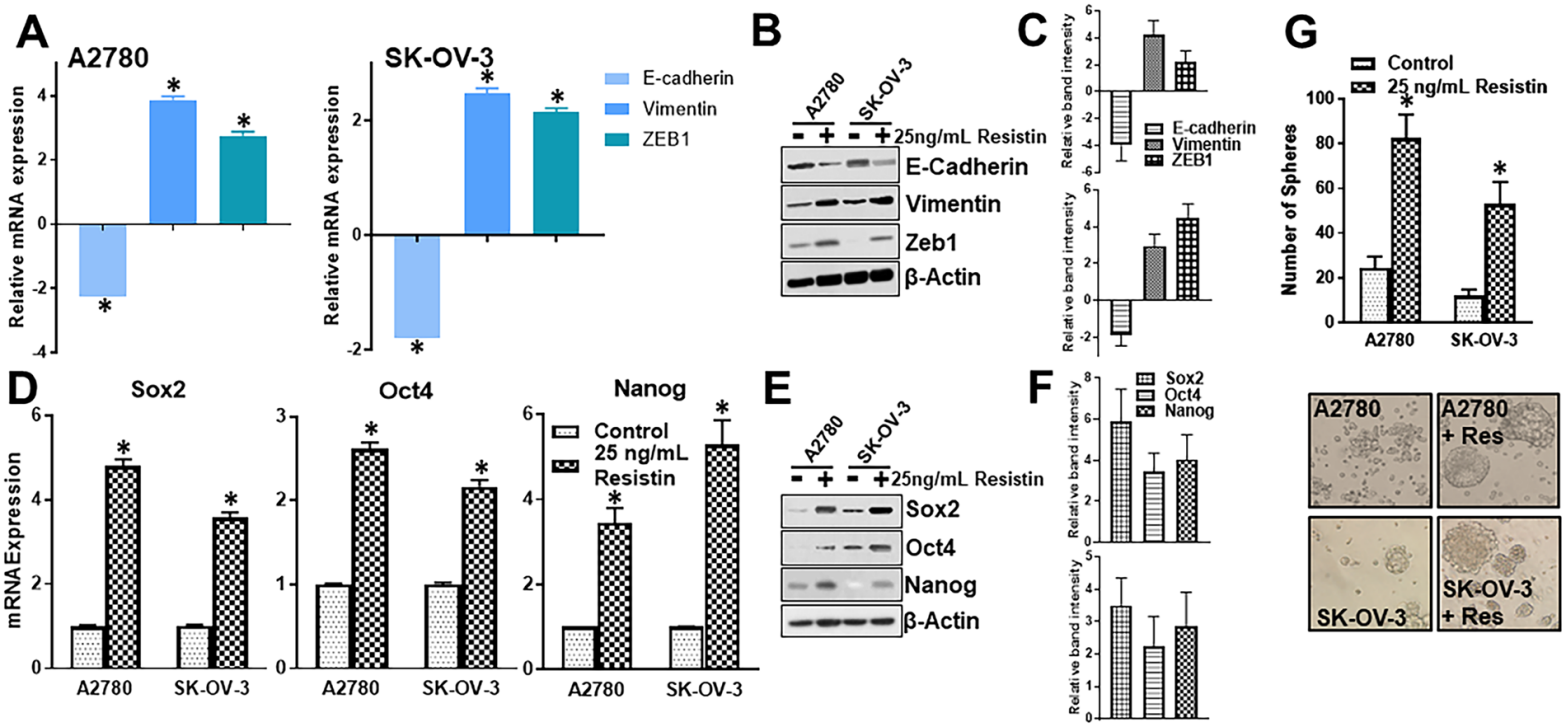
Resistin induces EMT and stemness in ovarian cancer cells. A2780 and SK-OV-3 cells were treated with resistin for 72 hours. (**A**) mRNA levels of EMT markers E-cadherin, vimentin and Zeb1 were evaluated by quantitative RT-PCR. The reported mRNA are relative to those in control (vehicle only treatment) which were set to 1 and the relative fold-changes in resistant-treated cells were calculated. (**B**) Protein levels were evaluated by western blotting and (**C**) relative intensity of western blot bands from three different repeats was calculated. Effect of resistin on induction of stemness was calculated by evaluating the effect on (**D**) mRNA and (**E**) protein expression levels of stemness markers sox2, oct4 and nanog. (**F**) Relative intensity of western blot bands from three independent repeats was calculated. (**G**) Stemness was determined by sphere formation assays. A2780 and SK-OV-3 cells were allowed to form spheres on ultra-low attachment plates in the presence of vehicle control or 25 ng/mL resistin. Representative pictures of spheres are shown below the respective bar graphs. *‘*+*Res’* indicates presence of added resistin. β-actin was used as loading control for western blots while GAPDH was used as internal control for RT-PCR analysis. *p < 0.01, compared to control.

To understand the underlying mechanisms by which resistin can induce invasion, cisplatin resistance Nand angiogenesis, we focused on the process of EMT. We evaluated levels of a few markers, such as e-cadherin, vimentin and ZEB1. E-cadherin is an epithelial marker while vimentin and ZEB1 are mesenchymal markers. We observed that resistin reduces the expression of e-cadherin by ~2-folds (p < 0.01) in both A2780 and SK-OV-3 (Fig. 4A) cells. This was accompanied by ~4-folds (p < 0.01) increase in vimentin and ~3-folds (p < 0.01) increase in ZEB1 in A2780 cells (Fig. 4A), and ~2-folds (p < 0.01) increase in vimentin and ZEB1 in SK-OV-3 cells (Fig. 4A). A similar effect was also observed on protein expression levels, as determined by western blotting (Fig. 4B,C). We also observed that resistin induces stemness in ovarian cancer cells. When we evaluated the mRNA and protein levels of three markers of stemness, sox2, oct4 and nanog, we found that resistin, at the tested dose of 25 ng/mL, increased mRNA transcript levels (Fig. 4D) as well as protein levels (Fig. 4E,F) of sox2, oct4 and nanog in both the cell lines. Stemness-inducing potential of resistin was conclusively determined through sphere-forming assay. We found that resistin induced the number of spheres, the surrogate markers for stem cells, in A2780 and SK-OV-3 cells (Fig. 4G). These results fully support EMT and stemness induction by resistin which can explain the resistin-mediated effects discussed above.

### Resistin effects are mediated by novel miRNAs

**Figure 5:**
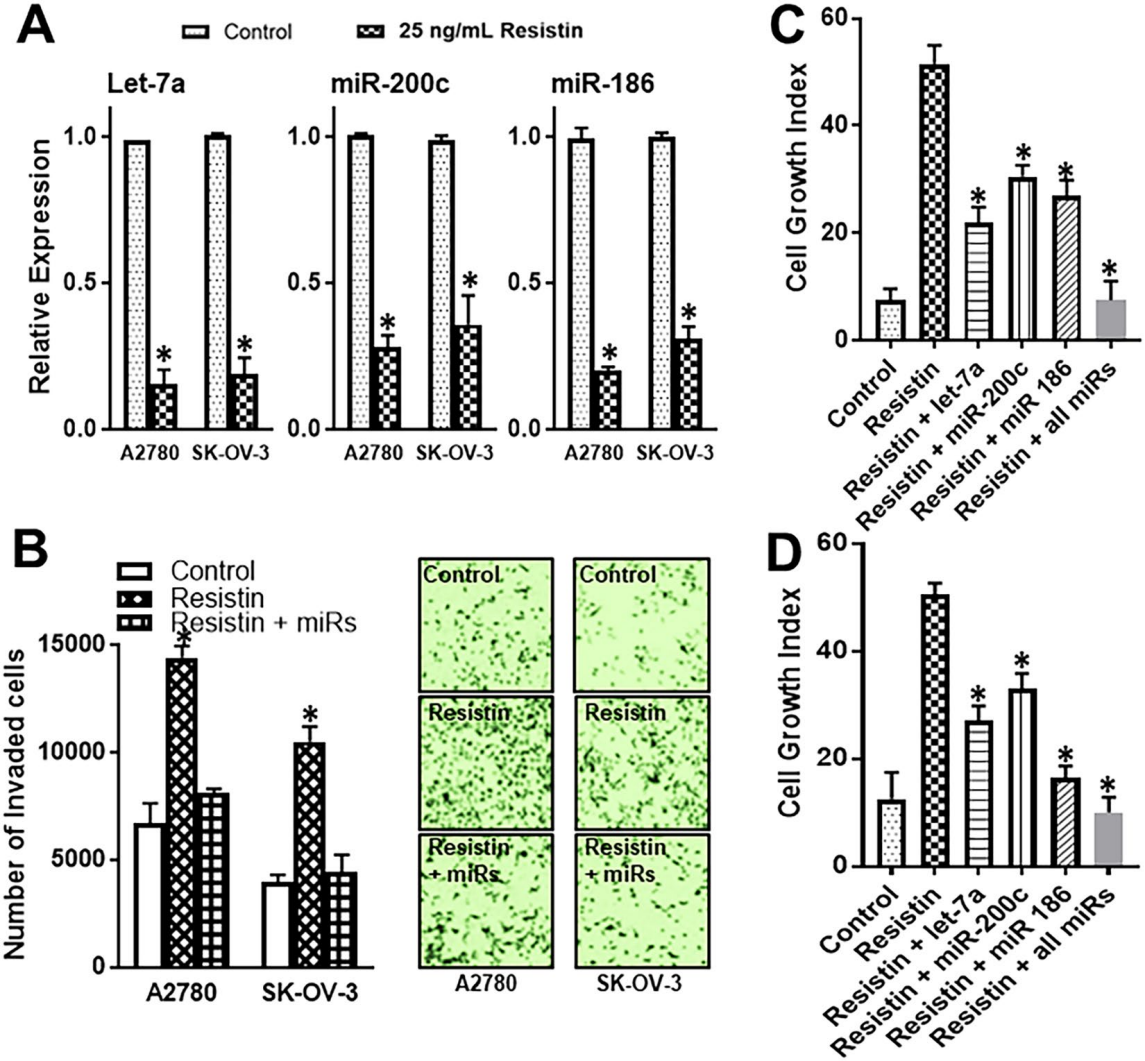
miRNAs abrogate resistin effects. (**A**) Levels of miRNAs (let-7a, miR-200c and miR-186) in vehicle and resistin (25 ng/mL) treated A2780 and SK-OV-3 ovarian cancer cells were measured by quantitative RT-PCR. U6 was used as internal control miRNA. *p < 0.01, compared to control. Cells were transfected with pre-miRs (let-7a, miR-200c, miR-186 or a combination of all three miRNAs). (**B**) Effect of such transfections was evaluated on resistin-mediated induction of invasion of A2780 and SK-OV-3 cells. Representative pictures from invasion assays are also shown. Further, the effects of miRNAs’ transfections on the growth of (**C**) A2780 and (**D**) SK-OV-3 cells in the presence of cisplatin (1.5 μM for A2780 cells and 15 μM for SK-OV-3 cells) for 72 hours were measured by MTT assay. The OD value for cells without cisplatin treatment was assigned a value of ‘100’ and the relative OD values of miRNA-transfected and resistin-treated cells are plotted to represent cell growth index. The ‘control’ refers to cells treated with just cisplatin. *p < 0.01, compared to resistin-treated cells.

EMT involves a functional role of several miRNAs. We screened a panel of miRNAs that have a proven role in induction of EMT. We found that a number of these miRNAs, particularly those belonging to let-7 and miR-200 family were controlled by resistin in individual cell lines (Results not shown). However, three miRNAs stood out, as they were consistently down-regulated by resistin in both the cell lines tested in this study. These were let-7a, miR-200c and miR-186 (Fig. 5A). To establish that regulation of these miRNAs is functionally relevant, especially to the induction of resistin-induced invasion, we transfected A2780 and SK-OV-3 cells with a combination of pre-let-7a, pre-miR-200c and pre-miR-186, and found that increased levels of pre-miRs abrogated the resistin-mediated invasiveness (Fig. 5B). Finally, we confirmed an involvement of miRNAs in the resistin-induced resistance to cisplatin. We checked the effect of individual miRNAs as well as the combination of all three miRNAs. Cells were treated with cisplatin in the presence of resistin and an effect of induced expression of miRNAs (through transfections with pre-miRNA oligonucleotides) was evaluated. As expected, we observed marked killing of cells by cisplatin (Fig. 5C,D control bars). Resistin, by itself, was able to significantly limit this effect of cisplatin. Further, re-introduction of miRNAs very significantly attenuated such resistin-mediated resistance in A2780 (Fig. 5C) as well as SK-OV-3 cells (Fig. 5D). Individual miRNAs significantly (p < 0.01) attenuated the resistin-mediated effects and a combination of all three miRNAs completely abolished the effects of resistin.

### *In vivo* validation of resistin effects

**Figure 6:**
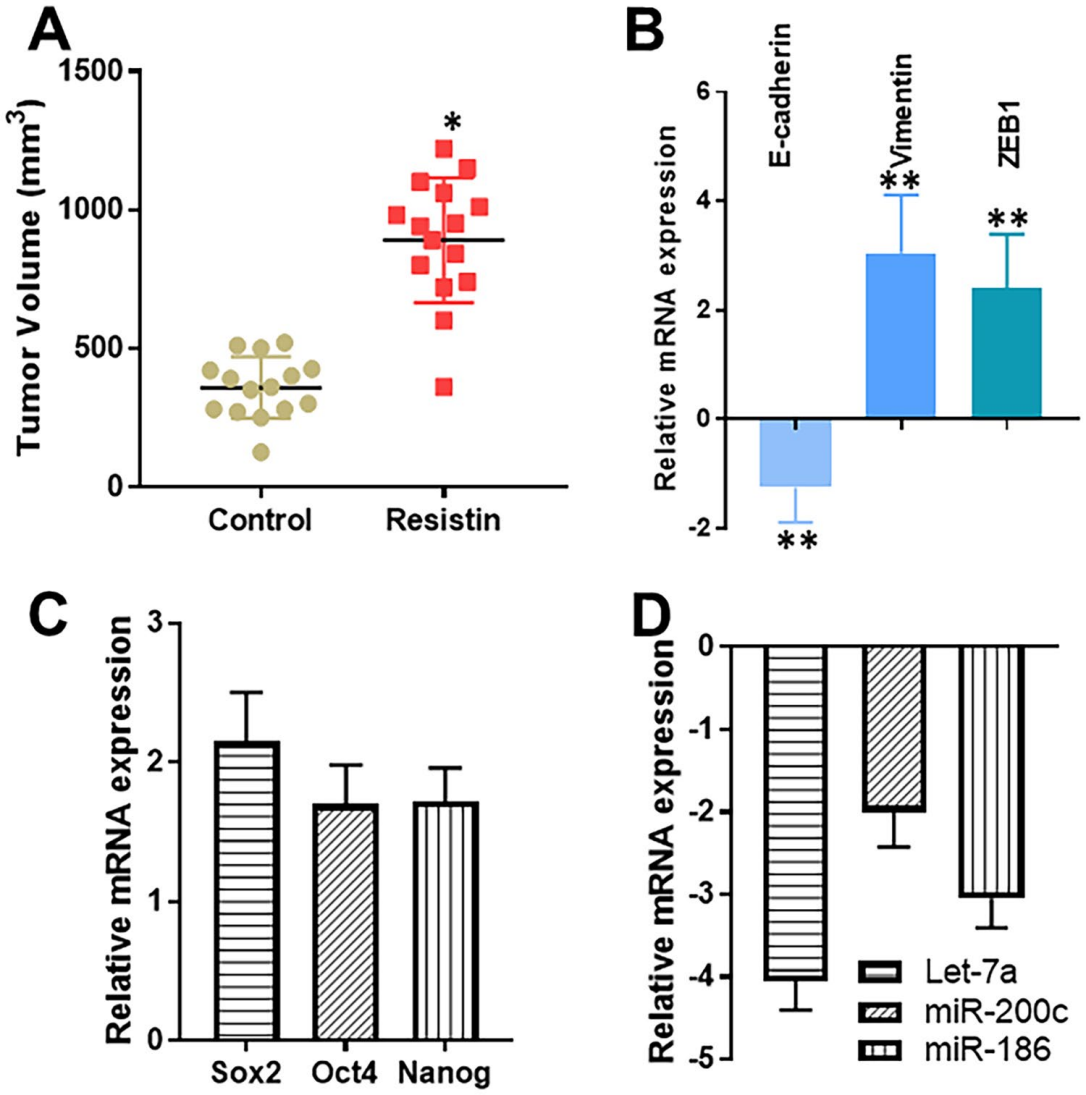
*In vivo* effects of Resistin. Ovarian cancer cells A2780 (2 million) were injected to establish xenografts in SCID mice. (**A**) Mice were administered resistin or PBS, as indicated in ‘Methods’, and tumor volumes calculated. (**B**) EMT markers (e-cadherin, vimentin and ZEB1), (**C**) stemness markers (sox2, oct4 and nanog) and (**D**) miRNA levels were quantitated by quantitative RT-PCR in tumor remnants. The shown values are relative to those in control mice (PBS-fed) which were set to 1 and the relative fold-changes were calculated. *p < 0.05 and **p < 0.01, compared to control.

Having established a definite role of resistin in ovarian cancer cells’ proliferation *in vitro*, we next sought to test the effect of resistin *in vivo*. For this, we established A2780 xenografts in nude mice (n = 10 for each group) and administered PBS (control) or resistin, via intravenous injections, to the mice, once tumors began to appear. Treatments continued for 3 weeks and the animals were sacrificed 2 weeks after the end of treatments. We observed a significantly increased tumor volume (p < 0.05) in the mice that were administered resistin (Fig. 6A). Also, since our *in vitro* results suggested an effect of resistin on EMT and stemness markers, we evaluated the levels of molecular markers of EMT and stemness in tumor remnants as well. We observed induced EMT (as evident by decreased e-cadherin and increased vimentin/ZEB, Fig. 6B) as well as stemness (as evident by increased sox2, oct4 and nanog, Fig. 6C). Further, the levels of miRNAs, let-7a, miR-200c and miR-186, were down-regulated in tumors of resistin-treated mice, relative to mice in the control group (Fig. 6D). These observations provide credible confirmation of our *in vitro* results, and confirm a role of resistin in the aggressiveness of ovarian cancer cells.

## Discussion

For many years, there have been attempts to connect resistin with polycystic ovary syndrome (PCOS), a hormone disorder with disturbed menstrual cycles and cysts on ovaries. While some investigations found no connection^18–20^, others suggested a link^21–24^. It has been proposed that high levels of resistin gene in adipocytes and the higher resistin secretion plays a role in PCOS^21,22^. Later on, a connection between resistin and human cancers was sought^25,26^. Resistin was investigated in breast cancer because of it’s adipocyte origin in murine models and the known functional connections between obesity and breast cancer. Resistin levels are reported to be significantly higher in breast cancer patients, in contrast to the control subjects^1^. Following this, another group of investigators found a connection between resistin and endometrial cancer risk^5^. This group revealed that the endometrial cancer patients have significantly higher levels of circulating resistin, relative to the control subjects. These reports relate importance of resistin levels in female cancers.

However, resistin is not just important for female cancers. In prostate cancer which only affects male populations, a research team found much higher resistin levels in high-grade cancer tissues^11^. Resistin was much lower in lower grade prostate cancers. The prostate cancer cell lines PC3 and DU145 expressed resistin which played a role in their proliferation. Another group of investigators saw a very similar trend in colorectal cancer as resistin levels were increased in colorectal cancer patients, relative to the controls^3^. A study conducted in pancreatic cancer revealed a very interesting role of resistin^27^. Resistin was reported to be important for relapse-free survival. Pancreatic cancer patients with negative resistin staining had double the relapse-free survival (18 months), in contrast to the patients whose tumors stained positive for resistin (9 months). In agreement with the findings of other research teams in other cancers, resistin levels were higher in high grade pancreatic cancers, as compared to the levels in the lower grade cancers. Similar to the pancreatic cancer study, another research team found a connection between resistin and relapse-free survival in breast cancer, and resistin associated with more advanced breast cancer^28^.

In ovarian cancer, there is a previous study investigating the role of resistin. This study focused on the effects of resistin on angiogenesis and the results suggested a dose- and time-dependent increase in the mRNA and protein expression of VEGF when ovarian cancer cells were treated with resistin^29^. In our study we found a similar increase in VEGF production. Our study employed A2780 and SK-OV-3 ovarian cancer cells while the previous study^29^ used HO-8910 cells. This means that the effect of resistin on VEGF production is not cell line-dependent. Our experiments provide an even stronger proof in support of angiogenesis stimulation by resistin because we show that, in addition to VEGF, resistin stimulates MMP2 production which is also a strong factor associated with angiogenesis. Further, we show that the effect of resistin is visible on mRNA transcripts as well as expression levels of VEGF and MMP2, with resulting increased secretions, thus establishing a significant contribution of resistin to angiogenesis. An earlier study in gastric cancer identified induction of CXCL12-CXCR4 signaling by resistin, which can be another mechanism of angiogenesis-induction^8^. Thus, through multiple mechanisms, resistin can induce angiogenesis in different cancers. Further, there is no report to-date reporting the levels of resistin in ovarian cancer patients, even though it is well regarded that resistin levels are generally elevated in cancer patients^9^. Serum levels of resistin in humans can vary over a broad range of 0.6 ng/mL to 27.7 ng/mL^30^. In disease conditions, such as cancer, the levels of resistin can be much elevated, correlating with the tumor stage. For example, in colon cancer, resistin levels have been reported to be as high as 46.4 ng/mL in Dukes’ B1/B2 stage colon cancer and as high as 87.6 ng/mL in Dukes’ C/D stage colon cancer^31^. Therefore, the resistin doses used in this study are clinically relevant.

An important finding of our study is the establishment of a role of resistin in EMT and stemness of ovarian cancer cells. This is the very first report for such effect of resistin in ovarian cancer cells, even though resistin was earlier reported to increase invasion, and the mesenchymal marker vimentin in breast cancer cells^32^. We not only observed an increase in vimentin in the ovarian cancer cells in our study, but further confirmed the EMT induction through increase in another mesenchymal marker, ZEB1, with a simultaneous decrease in the epithelial marker, e-cadherin. Further, we observed the induction at mRNA as well as expression level, thus providing credibility to our observations. Cancer cells that undergo EMT are more invasive. Additionally, EMT, stemness and invasive potential are mechanistically connected. Accordingly, our observations on induction of stemness markers sox2, oct4 and nanog by resistin further support the induction of invasiveness by resistin. Thus our results about increased invasion in resistin-treated cells are probably connected to EMT and stemness induction. As a further mechanism, we demonstrate an essential involvement of three EMT-inducing miRNAs, let-7a, miR-200c and miR-186 in mediating resistin effects. Since we identified EMT as a key mechanism of resistin action, we initially performed a screening of miRNAs with a focus on those miRNAs that have been reported to play a role in EMT. All of these three shortlisted miRNAs are reported inhibitors of EMT and, therefore, their expression was induced in ovarian cancer cells, through the use of pre-miRNAs. This resulted in attenuation of resistin-mediated effects on invasion of A2780 and SK-OV-3 cells.

We also report that resistin induces resistance against chemotherapy in ovarian cancer cells. Our results show that resistin, dose-dependently, induces significant resistance against cisplatin. An interesting observation was that resistin could not only induce cisplatin-resistance in the parental A2780 cells, but also in the A2780Cis cells. A2780Cis were made resistant to cisplatin by continuous exposure to cisplatin. It is well known that cells acquire resistance against chemotherapy through many different mechanisms. It is possible that the mechanism of cisplatin resistance in A2780Cis cells is distinct from the mechanism activated by resistin. Therefore, resistin was able to further augment the resistance to cisplatin even in these resistant cells. Our results on chemoresistance are the first reports in ovarian cancer cells, but complement similar findings by other research teams, such as reported role of resistin in inducing resistance against doxorubicin in breast cancer cells^13,14^, multiple drug resistance in myeloma^15^ and 5-fluorouracil resistance in colon cancer cells^33^. The observed resistance against cisplatin can be explained by our results that resistin increases stemness in ovarian cancer cells. Interestingly, very recently a report has emerged which establishes a role of resistin in inducing EMT and stemness of breast cancer cell, which it accomplishes by being a ligand for toll-like receptor-4 (TLR4), and by stimulating NF-κB-STAT3 signaling^34^. Our results assume further significance because we present *in vivo* data to support *in vitro* findings. We show that resistin supports tumor growth in an *in vivo* mouse model with data suggesting induction of EMT and stemness markers in tumor remnants.

In summary, we report, for the very first time, a role of resistin in invasion and chemoresistance of ovarian cancer cells. We establish a mechanistic role of miRNA-regulated EMT in the chemoresistance, and, thus, establish resistin as a potentially important target for possible intervention in advanced stage and relapsed ovarian cancers.
